# Percentage of small platelets on peripheral blood smear and Child-Turcott-Pugh class can predict the presence of oesophageal varices in newly diagnosed patients with cirrhosis: development of a prediction model for resource limited settings

**DOI:** 10.1186/s12876-019-1054-5

**Published:** 2019-07-26

**Authors:** K. Perera, S. K. Kodisinghe, D. S. Ediriweera, D. Moratuwagama, S. Williams, A. Pathmeswaran, M. A. Niriella, H. J. de Silva

**Affiliations:** 1 Colombo North Teaching Hospital, Ragama, Sri Lanka; 2 Faculty of Medicine, University of Kelaniya, Ragama, Sri Lanka

**Keywords:** Oesophageal varices, Varices, Cirrhosis, Prediction, Haematology, Small platelets, Child class

## Abstract

**Background:**

In cirrhosis upper-gastrointestinal-endoscopy (UGIE) identifies oesophageal varices (OV). UGIE is unavailable in most resource-limited settings. Therefore, we assessed prediction of presence of OV using hematological parameters (HP) and Child-Turcott-Pugh (CTP) class.

**Methods:**

A prospective study was carried out on consecutive, consenting, newly-diagnosed patients with cirrhosis, in the University Medical Unit, Colombo North Teaching Hospital, Ragama, Sri Lanka from April 2014–April 2016. All patients had UGIE to evaluate presence and degree of OV, prior to appropriate therapy. HP (full blood count with indices using automated analyzer and peripheral blood smear using Leishmann stain) and CTP class were assessed on admission. Linear logistic regression model was developed to predict OV using HP and CTP class.

**Results:**

54-patients with cirrhosis were included [14(26%), 24(44%) and 16(30%) belonged to CTP class A, B and C respectively]. 37 had varices [CTP-A 4/14(26.6%), CTP-B 19/24(79.2%), CTP-C 14/16(87.5%)] on UGIE. Generalized linear model fitting showed decreasing percentage of small platelets (%SP) (*P* = 0.002), CTP-B (*P* = 0.003) and CTP-C (P = 0.003) compared to CTP-A had higher probability of having OV. The model predicts the log odds for having OV = − 0.189 – (0.046*%SP) + 2.9 [if CTP-B] + 3.7 [if CTP-C]. Based on receiver operating characteristic (ROC) analysis, a model value > − 0.19 was selected as the cutoff point to predict OV with 89%-sensitivity, 76%-specificity, 89%-positive predictive value and 76%-negative predictive value.

**Conclusions:**

We constructed a model using %SP on peripheral blood smear and CTP class. This model may be used to predict the presence of OV, in newly diagnosed patients with cirrhosis, with acceptable sensitivity and specificity, to prioritize the patients who deserve early UGIE in limited resource settings.

**Electronic supplementary material:**

The online version of this article (10.1186/s12876-019-1054-5) contains supplementary material, which is available to authorized users.

## Background

Oesophageal varices (OV) are a major complication of cirrhosis secondary to the development of clinically significant portal hypertension [1]. OV can lead to life threatening bleeding, further decompensation and death among patients with cirrhosis. Identifying patients with cirrhosis who are at risk of bleeding from OV is important to prevent overt bleeding, improve survival and minimize health care costs [2]. This will help to minimize the burden on emergency upper gastrointestinal endoscopy (UGIE) services, admissions to high-dependency units, use of expensive pharmacological agents, utilization of blood and blood product support and further decompensation of these patients resulting in prolong hospital stay.

To detect OV, it is recommended to screen all the patients with newly diagnosed cirrhosis by UGIE as per current clinical practice guidelines [2]. The aim of the UGIE screening is to identify those patients who are at increased risk of bleeding from OV and to select those who should receive primary preventive prophylactic treatment [non-selective β-block therapy or endoscopic variceal ligation (EVL)] [1, 2].

Although the gold standard of detecting OV is UGIE, this method of screening is not readily available in the health care centers in resource limited settings. In the past, several studies have attempted to assess presence of OV by non-invasive means other than UGIE, utilizing clinical assessments, biochemical parameters, splenic diameter by ultrasound scan, platelet count to spleen diameter ratio, transient elastography (FibroScan) and computer tomography scans [3–10]. However, none of those could show non-inferiority of UGIE which is still the gold standard.

Recently it has been suggested that the use of non-invasive liver stiffness (LS) measured by transient elastography (TE) (FibroScan) combined with the platelet count can be used to exclude clinically significant portal hypertension and presence of OV [11]. Although this non-invasive assessment excludes the need for an more invasive UGIE in selected patients with cirrhosis, it is not readily available in most centers. Therefore, a simpler, non-invasive and more accessible method to predict the presence and the degree of OV is needed for resource poor setting. Utilizing an already available, cost effective, non-invasive, routine investigation to predict OV will be useful for primary care setting to triage, stratify and arrange early referral for those patients who are at a higher risk of OV to centers with facilities and expertise for UGIE.

Sri Lanka is a developing country with resource limited settings to manage patients with cirrhosis and its complications. In Sri Lanka the most common causes of cirrhosis are alcohol related and cryptogenic [probably non-alcoholic steatohepatitis (NASH)-related], whereas chronic hepatitis B & C infections and haemachromatosis are uncommon aetiologies for cirrhosis [12]. NASH-related cirrhosis is rapidly increasing in Sri Lanka and is the dominant cause for liver transplantation [13].

In the present study, we attempted to assess the associations and the utility of changes in haematological parameters in the full blood count (FBC) and peripheral blood morphology and the Child-Turcotte-Pugh (CTP) class, among patients with cirrhosis, as a useful predictors of presence and degree of OV in our resource limited setting. A FBC and a peripheral blood smear are ready available resources. Identifying the morphological changes in a peripheral blood smear only requires a well stained blood smear and trained non-specialist medical officer in a resource poor setting.

## Methods

### Study design, study setting, study population

This was a prospective-study conducted at the University Medical Unit, Colombo North Teaching Hospital, Ragama, Sri Lanka from April 2014 to April 2016. All biochemically and radiologically proven, newly diagnosed patients with cirrhosis, who were not on treatment or prophylaxis for OV were screened for eligibility. Out of them, 54 consecutive, consenting patients with complete data were included in the study. They were divided an included in three groups based on the CTP score: CTP class A (score 5-6), CTP class B (scores 7-9) and CTP class C (scores 10-15).

Cirrhosis was diagnosed on clinical, biochemical, ultrasonographic criteria in most cases. The infrequent, inconclusive cases when possible or required were confirmed by liver biopsy. Patients with cirrhosis who were already on prophylactic or therapeutic drugs (β blockers – propranolol, carvedilol; diuretics - furosemide, spironolactone; metronidazole) or had undergone EVL were excluded from the study. Patients with cirrhosis with confounding factors for thrombocytopenia such as concurrent viral or severe bacterial infection, autoimmune diseases & chronic disseminated intravascular coagulation were also excluded from the study population using clinical findings, FBC & blood picture and other laboratory investigations such as C-reactive protein, erythrocyte sedimentation rate, antinuclear antibody and coagulation profile wherever necessary.

### Sample calculation

To calculate the sample size, we assumed the following assumptions at the time of diagnosis: 50% of cirrhotic patients to have OV and 50% do not have OV; among them, the prevalence of expected haematological parameters in those without OV is 40% and the prevalence of expected haematological parameters in those with OVs is 80%. Therefore, a total sample size of 46 (with and without OV) will have 80% power to detect this difference at 5% level of significance.

### Method of data collection

An interviewer administered questionnaire was used to record demographic, clinical, biochemical and ultrasonographic parameters, CTP class, UGIE findings & haematological parameters.

- Assessment of esophageal varices by UGIE

All patients underwent diagnostic UGIE to document the presence or absence as well as the grading of OV according to variceal size: small OV with < 5 mm in size with minimal elevation from the mucosal surface or large OV > 5 mm in size involving more than one third of the esophageal mucosa, according to the World Gastroenterology Organization practice guidelines on OV [14].

b) Assessment of haematological parameters and peripheral blood morphology

Two ml of ethylene diamine tetra-acetic acid (EDTA) anticoagulated blood was collected prior to any transfusions and processed by an automated hematology analyzer in order to obtain the following hematological readings: Total white blood count (WBC), differential leukocyte count (DC), hemoglobin (Hb), packed cell volume (PCV), red cell count (RCC), red cell distribution width (RDW), mean corpuscular volume (MCV), Mean corpuscular hemoglobin (MCH), mean corpuscular hemoglobin concentration (MCHC), platelet count and mean platelet volume (MPV).

Peripheral blood smears were stained by Leishman stain. The blood films were assessed by a Consultant Haematologist, blinded for clinical and endoscopic findings of the patients, for the presence of target cells, macrocytes, fragmented cells and other abnormalities. In addition, assessment of visual platelet count was done in order to overcome possible errors with the automated haematology analyzer. The platelet size was assessed morphologically and divided into three groups: small, normal and large and giant size. Small platelets were defined as nearly pinpoint, large platelets defined as closer towards red cell size and normal size defined as in between small and large.

### Data analysis

The haematological indices in the FBC and peripheral blood morphological characteristics were compared between patients with and without OV using Student’s t test. Haematological indices in the FBC and peripheral blood morphological characteristics which showed differences between the two groups were then assessed to determine the association with the size of the OV using Student’s t test.

Generalized linear models were used to model the presence and absence of OV among patients. Initially exposure variables were screened for presence of OV and subsequently the exposure variables which were significant at *P* = 0.2 were considered for model fitting. Generalized additive models were used to assess the non-linear association between exposure variables and presence of OV. Goodness of the fitted models were assessed with standardized residuals analysis and deviance test. *P* value of < 0.05 was considered as significant. Receiver operating characteristic (ROC) analysis was used to determine the model cutoff point for presence and absence of OV. Analysis was done with R programming language version 3.5.1.

### Ethical issues and clearance

The ethical clearance for the study (P/076/04/2014) was obtained from the Ethics Review Committee, Faculty of Medicine, University of Kelnaiya, Ragama, Sri Lanka. The purpose of the study was explained and voluntary participation was ensured in obtaining informed written consent from all the participants.

## Results

Fifty four patients [46 (85.2%) males and 8 (14.8%) females] with complete data out of 77 newly diagnosed patients with cirrhosis were included. Twenty three patients were not included in the analysis because of multiple reasons such as death, defaulted follow up, long waiting list, being unable to have UGIE, missing blood films and having blood transfusions before collecting the sample for FBC and blood film.

Majority (28.6%) of the patients was in the over 60 year age group and only 9.1% were below the age of 40 years. Out of 54 patients with cirrhosis 14 (26%), 24 (44%) and 16 (30%) belonged to CTP class A, B and C respectively. In the study sample, 37/54 (68.5%) had OV on UGIE. Out of them, 4/14 (26.6%), 19/24 (79.2%), and 14/16 (87.5%) were in Child class A, B, and C respectively (Table 1).

**Table 1:** Characteristics of the study population

Characteristic	OV absent *N* = 17 (31.5%)	OV present *N* = 37 (68.5%)
Age group (years)		
30–39	3 (17.6%)	4 (10.8%)
40–49	4 (23.5%)	11 (29.7%)
50–59	3 (17.6%)	7 (18.9%)
> 60	7 (41.2%)	15 (40.5%)
Number of males (%)	12 (70.6%)	34 (91.9%)
Aetiology of Cirrhosis		
Alcohol related	7 (41.2%)	17 (45.9%)
Crypotogenic (NASH-related)	10 (58.8%)	20 (54.1%)
CTP Class		
A	10 (58.8%)	4 (10.8%)
B	5 (29.4%)	19 (51.4%)
C	2 (11.8%)	14 (37.8%)

Patients with OV of any size showed low red cell count (3.40 vs 3.95, *P* = 0.019), high MCV (97.14 vs 91.63, *P* = 0.04), high RDW (17.74 vs 15.41, *P* = 0.005) and thrombocytopenia (automated count 115 vs 188, *P* = 0.008) and high MPV (8.9 vs 7.9, *P* value 0.019). However, automated white cell count and differential counts did not show difference with presence or absence of OV (Table 2).

**Table 2:** Haematological parameters (FBC and indices) between presence and absence of OV

Haematological parameter*	Mean (SD) in patients without OV *N* = 17 (31.5%)	Mean (SD) in patients with OV *N* = 37 (68.5%)	*P* value**
WBC count (per mm^3^)	8500 (2264)	8750 (3849)	0.769
% neutrophils	58.3 (13.2)	60.9 (20.7)	0.569
% lymphocytes	34.1 (14.9)	29.2 (18.1)	0.309
% eosinophils	3.9 (3.6)	5.4 (4.9)	0.219
% monocytes	3.8 (3.6)	3.7 (3.1)	0.973
% basophils	0.0 (0.0)	0.3 (0.9)	0.077
RCC (10^12^/L)	**3.9 (0.8)**	**3.4 (0.6)**	**0.019**
Hb (g/dL)	11.5 (2.3)	10.5 (1.8)	0.117
Hct (%)	36.0 (7.0)	32.6 (5.8)	0.093
MCV (fl)	**91.6 (8.2)**	**97.1 (10.1)**	**0.040**
MCH (pg)	29.3 (3.7)	31.1 (3.8)	0.119
MCHC (%)	31.9 (1.7)	32.1 (1.9)	0.712
RDW (%)	**15.4 (2.4)**	**17.7 (3.1)**	**0.005**
Platelet count (10^9^/L) (automated)	**188 (92.4)**	**115 (71.1)**	**0.008**
Platelet count (10^9^/L) (visual)	**195 (88.1)**	**129 (69.4)**	**0.011**
MPV (fL)	**7.9 (1.2)**	**8.9 (1.5)**	**0.019**

*Note: all are automated count unless specified

**Student’s t test (2 tail)

Bold - Statistically significant variables

Table 3 shows the RBC morphological characteristics of patients. Number of macrocytes (36% vs 15%, *P* = 0.02) and acanthocytes (2% vs 1%, *P* = 0.04) were high in patients with any size of OV. Mean percentage of small platelets were higher in patients who did not have OV compared with those with OV (40% versus 13% respectively, *P* = 0.006) (Table 3). None of the FBC indices and peripheral blood morphology showed significant differences between small versus large OV (Table 4).

**Table 3:** The peripheral blood morphology between presence and absence of OV

Morphology		Mean % (SD) of cells in patients without OV *N* = 17 (31.5%)	Mean % (SD) of cells in those with OV *N* = 37 (68.5%)	*P* value
RBC	Normochromic normocytic	71.0 (34.3)	51.7 (30.7)	0.058
	Hypochromic microcytic	5.0 (14.6)	2.9 (5.7)	0.583
	Pencil cells	0.4 (1.4)	0.3 (0.9)	0.823
	Target cells	4.5 (17.6)	5.0 (9.5)	0.913
	Tear drop cells	2.5 (2.9)	1.0 (1.8)	0.058
	**Macrocytes**	**15.2 (26.0)**	**36.0 (33.8)**	**0.018**
	**Acanthocytes**	**0.9 (1.1)**	**2.0 (2.6)**	**0.044**
	Partially haemoglobinized cells	0.01 (0.05)	0.15 (0.39)	0.047
	Contracted cells	0.1 (0.2)	0.2 (0.5)	0.483
	NRBCs	0.01 (0.05)	0.02 (0.06)	0.766
	Polychromatic cells	0.5 (1.4)	0.5 (1.1)	0.957
	Stomatocytes	0.06 (0.2)	0.12 (0.4)	0.495
	Spherocytes	0.0 (0.0)	0.2 (0.8)	0.187
	Fragments	0.08 (0.25)	0.07 (0.33)	0.882
Neutrophil	Normal morphology	54.8 (12.0)	54.1 (17.8)	0.863
	Neutrophils with Toxic granules	0.06 (0.24)	0.49 (1.82)	0.169
	Neutrophils with cytoplasmic vacuoles	0.3 (0.5)	0.4 (0.5)	0.551
	Band forms	0.7 (1.7)	1.4 (2.7)	0.255
	Myelocytes	0.06 (0.24)	0.19 (0.51)	0.195
	Blasts	0.0 (0.0)	0.0 (0.0)	
	Hypersegmented Neutrophils	3.2 (3.8)	5.1 (8.2)	0.239
	Reactive lymphocytes	0.3 (0.6)	0.6 (2.2)	0.364
Platelet size	**Normal**	**57.8 (31.8)**	**82.6 (21.1)**	**0.008**
	Large	2.1 (4.1)	4.3 (7.3)	0.165
	Giant	0.02 (0.07)	0.12 (0.31)	0.085
	**Small**	**40.1 (33.8)**	**12.9 (21.0)**	**0.006**

Bold - Statistically significant variables

**Table 4:** Haematological parameters (FBC with indices) and peripheral blood morphology between cirrhotics with small versus large OV

Significant parameters in Cirrhotic patients with OV	Mean (SD) in cirrhotics with Small OV 24 (31.5%)	Mean (SD) in cirrhotics with Large OV 13 (68.5%)	P value
RCC (10^12^/L)	3.35 (0.74)	3.50 (0.41)	0.422
MCV (fL)	94.91 (9.95)	101.25 (9.46)	0.067
RDW (%)	17.92 (3.22)	17.39 (2.83)	0.610
Platelet Count (10^9^/L) (automated)	118.88 (80.06)	107.54 (52.93)	0.609
Platelet Count (10^9^/L) (visual)	131.25 (75.49)	124.69 (59.06)	0.773
MPV (fL)	9.09 (1.81)	8.44 (0.95)	0.160
% macrocytes	31.92 (32.19)	43.54 (36.71)	0.348
% acanthocytes	2.21 (3.13)	1.52 (1.61)	0.385
% of small platelets	10.38 (13.03)	17.62 (30.92)	0.433

### Prediction model

**Fig. 1:**
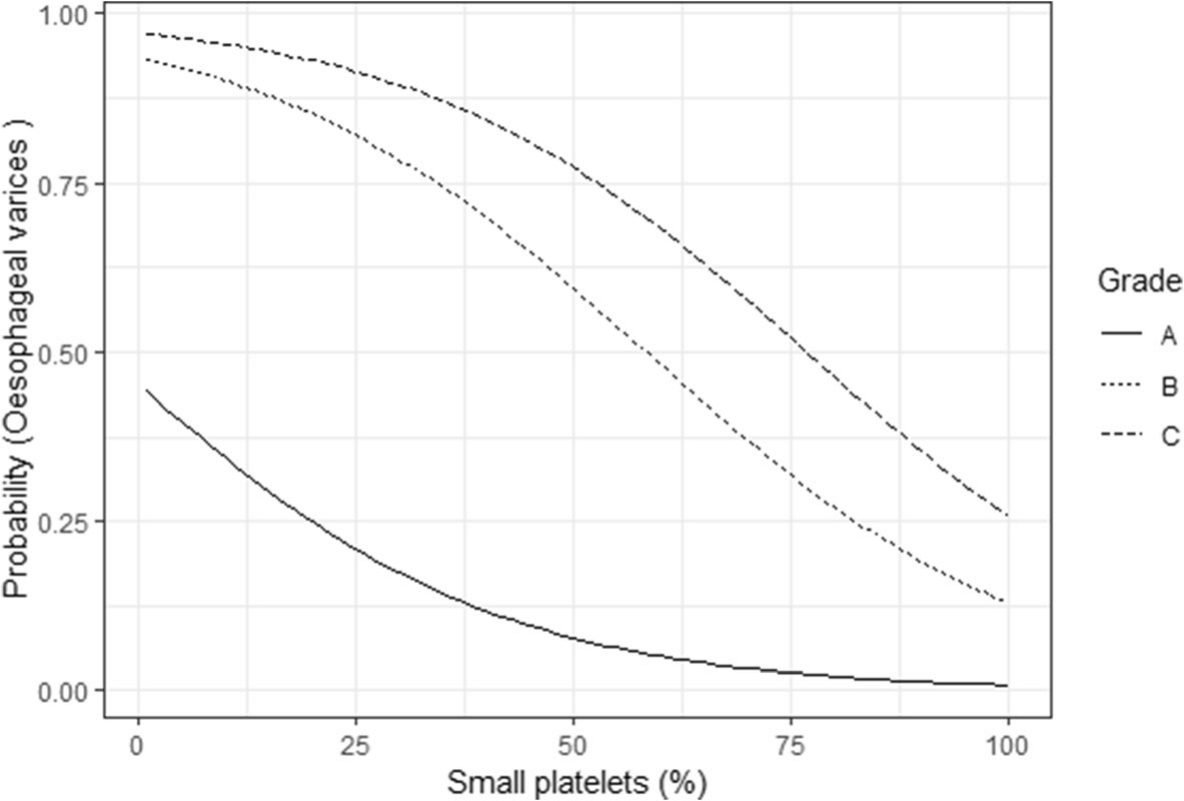
Probability of having OV along with the number of small platelets for each CTP class

The fitted multiple linear logistic regression model to predict presence and absence of OV is given in Table 5. The model did not show extrabinomial variation (residual deviance: 40.2 on 50 degrees of freedom, *P* < 0.05) and was considered as the final model for prediction. According to the model, percentage of small platelets (%SP) (Odds ratio = 0.96, 95% CI: 0.93–0.98, *P* = 0.002), CTP class B compared to class A (Odds ratio = 17.32, 95% CI: 2.72–110.13, *P* = 0.003) and CTP class C compered to class A (Odds ratio = 40.24, 95% CI: 3.61–447.90, P = 0.003) showed significant association with the presence of OV (Fig. 1).

**Table 5:** Fitted linear logistic regression model to predict the probability of having OV on screening UGIE

Variable	Estimate	Std error	Z value	*P* value
Intercept	−0.189	0.652	−0.290	0.771
Small platelet count	−0.046	0.015	−0.310	0.002
CTP class B (compered to Class A)	2.852	0.944	3.021	0.003
CTP class C (compered to Class A)	3.695	1.229	3.005	0.003

The prediction formula;

$$ {\displaystyle \begin{array}{c}\mathrm{Log}\ \mathrm{odds}\ \left(\mathrm{presence}\ \mathrm{of}\ \mathrm{OV}\right)=\hbox{-} 0.189\\ {}\hbox{-} 0.046\ast \%\mathrm{SP}\\ {}+2.9\ \left[\mathrm{if}\ \mathrm{C}\mathrm{TP}\ \mathrm{class}\ \mathrm{B},\mathrm{otherwise}\ \mathrm{zero}\right]\\ {}+3.7\ \left[\mathrm{if}\ \mathrm{C}\mathrm{TP}\ \mathrm{class}\ \mathrm{C},\mathrm{otherwise}\ \mathrm{zero}\right]\end{array}} $$

Above formula provides the log odds for having OV for a given patient. Formula requires %SP of the patient and an addition of a constant based on the relevant CTP class (i.e. none for CTP class A, 2.9 for CTP class B, 3.7 for CTP class C). Based on ROC curve, a model predicted value of − 0.19 was selected as the cutoff point to determine the presence and absence of OV (Additional file 1: Figure S1). Predicted model showed 89% sensitivity and 76% specificity (Table 6). The positive predictive value (PPV) and negative predictive values (NPV) were 89 and 76% respectively. Area under the curve of ROC was 0.88. Based on this proposed model, a calculated rounded value > 0.0 predicts absence of OV, while a calculated rounded value < 0.0 predicts the presence of OV.

**Table 6:** Model prediction versus presence and absence of OV

	Varices - yes	Varices – no	Row total	
Model – yes	33	4	37	PPV = 89.2%
Model – no	4	13	17	NPV = 76.5%
Column total	37	17		
	Sensitivity = 89.2%	Specificity = 76.5%		

*PPN* Positive predictive value, *NPV* Negative predictive value

## Discussion

We constructed a model-based formula to predict presence of OV among the newly diagnosed patients with cirrhosis using the %SP and CTP class. Reduced %SP on peripheral blood smear, CTP class B and C were the independent predictors for presence of OV. Our model value > − 0.19 cutoff point to predict OV showed acceptable sensitivity (89%), specificity (76%), PPV (89%) and NPV (76%). A calculated rounded value < 0.0 predicted the presence of OV. FBC and indices obtained by automated analyzer and morphology of red cells and white cells examined on peripheral blood film did not show any predictability of the presence of OV. The size of varices could also not be predicted by any of the tested haematological parameter or the CTP class.

In the present study, having a higher %SP was protective for the presence of OV at screening UGIE regardless of their CTP class. It was more predictive in those with CTP class B or C. However, even among patients with CTP class A - compensated cirrhosis, having low %SP on blood film examination was predictive of the presence of OV. Therefore, newly diagnosed patients with low %SP should be prioritized for screening UGIE irrespective of the CTP class. In clinical practice, patients with CTP class C are usually given priority to be high up in the list to have UGIE than the CTP class A and B. Therefore, this prediction model gives an important clues to prioritize the CTP class A and B patients to have screening UGIE according to the %SP determined by blood film.

The size of OV could not be predicted by the %SP or the CTP class. This may be that %SP only correlates with the appearance of clinical significant portal hypertension (CSPH) hence the presence of OV. %SP may not necessarily correlate with the progression of CSPH which is reflected by absence of correlation with the size of OV.

The most accepted non-invasive screening method for the exclusion of the presence and the need for UGIE in patients with cirrhosis in the use of combination of LS assessed by TE and the platelet count. A combination LS < 20 kPa and platelet count > 150,000/mm^3^ is indicative of absence of oesophageal varices according to the latest guidelines [11]. However, LS measurements by TE are not routinely available in the resource limited setting making this recommendation less implementable in most countries with emerging economies. In contrast, the proposed model for the prediction of OV from the present study, only utilize the features from a peripheral blood smear and biochemistry necessary to calculate the CTP score. Therefore, we feel this is ideal for use in the resource limited setting to triage patient with cirrhosis for UGIE.

We expected thrombocytopenia and possibly MPV could predict the presence of OV based on previously published literature. Interestingly in our study, even with precautions were taken to collect data on confounding factors of thrombocytopenia such as patient comorbidities, drugs causing thrombocytopenia and complications of cirrhosis, we could not find independent predictive value of the platelet count for the presence of OV on screening UGIE. This may have been due to the relatively small sample size in the present study. Recruiting a larger sample of newly diagnosed patients with cirrhosis, who were not commenced on any therapeutic or prophylactic measures, from a single center was difficult. A multi-center study would have alleviated this problem.

There were several limitations of this study. On blood film examination, we counted platelets as large, small and normal based on visual judgement to calculate the percentage of platelets according to the size difference. The size of the platelets was determined only by visual inspection by the heamatologist and may have been subjective. We did not have a facility to measure PDW (Platelet distribution width) on our automated analyzer. PDW might have had a predictive significance of OV. Furthermore, the findings of this study were not validated in an independent cohort of patients with newly diagnosed cirrhosis. Therefore, the suggested predictive model can only be recommended for clinical use after its validation in an independent external cohort.

## Conclusions

In conclusion, reduced %SP examined on peripheral blood smear along with CTP class B or C can predict the presence of OV in newly diagnosed patients with cirrhosis with acceptable sensitivity and specificity. Therefore, the proposed model and the cutoff may be used in a resource limited setting to triage patients with cirrhosis for UGIE.

## Additional file

Additional file 1: **Figure S1.** Receiver operating Characteristic (ROC) curve to estimate the diagnostic accuracy of the prediction model. (PNG 12 kb)

## Data Availability

The de-identified datasets used and analyzed during the current study are only available from the corresponding author on prior request, after notification to and approval of the ERC, Faculty of Medicine, University of Kelaniya, Sri Lanka.

## Abbreviations

%SP: Percentage of small platelets
CSPH: Clinically significant portal hypertension
CTP: Child-Turcotte-Pugh
DC: Differential leukocyte count
EDTA: Ethylene diamine tetra-acetic acid
EVL: Endoscopic variceal ligation
FBC: Full blood count
Hb: Hemoglobin
LS: Liver stiffness
MCH: Mean corpuscular hemoglobin
MCHC: Mean corpuscular hemoglobin concentration
MCV: Mean corpuscular volume
MPV: Mean platelet volume
NASH: Non-alcoholic steatohepatitis
NPV: Negative predictive value
OV: Oesophageal varices
PCV: Packed cell volume
PDW: Platelet distribution width
PPV: Positive predictive value
RCC: Red cell count
RDW: Red cell distribution width
ROC: Receiver operating characteristic
TE: Transient elastography
UGIE: Upper gastrointestinal endoscopy
WBC: White blood count
