# Supplementary figures and images for: Percentage of small platelets on peripheral blood smear and Child-Turcott-Pugh class can predict the presence of oesophageal varices in newly diagnosed patients with cirrhosis: development of a prediction model for resource limited settings
Source: BMC Gastroenterol. 2019 Jul 26;19:134. doi: 10.1186/s12876-019-1054-5 (PMC6660923; doi:10.1186/s12876-019-1054-5)

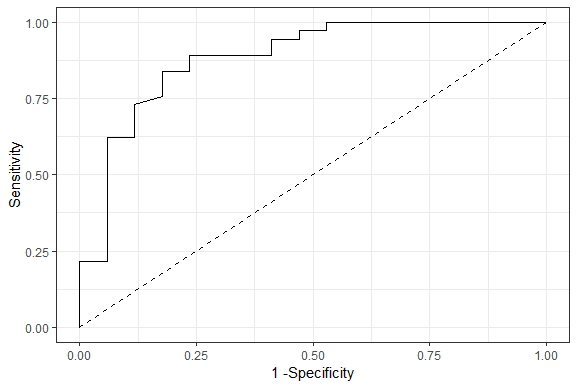

Supplement: Supplementary file 1 — Figure S1. Receiver operating Characteristic (ROC) curve to estimate the diagnostic accuracy of the prediction model. (PNG 12 kb) [file 12876_2019_1054_MOESM1_ESM.png]
